# MicroRNA profile in HBV-induced infection and hepatocellular carcinoma

**DOI:** 10.1186/s12885-017-3816-1

**Published:** 2017-12-01

**Authors:** Guanyu Wang, Fulu Dong, Zhiyao Xu, Sherven Sharma, Xiaotong Hu, Dafang Chen, Lumin Zhang, Jinping Zhang, Qinghua Dong

**Affiliations:** 1 Department of General Surgery, Sir Run Run Shaw Hospital, School of Medicine, Zhejiang University, Hangzhou, Zhejiang China; 2 Institutes of Biology and Medical Sciences, Soochow University, Soochow, Jiangsu, China; 3 Key Lab of Biomedical Research Center, Sir Run Run Shaw Hospital, School of Medicine, Zhejiang University, Hangzhou, Zhejiang China; 4 David Geffen School of Medicine at UCLA, and the Department of Veterans Affairs, Los Angeles, CA USA; 5 Key Laboratory of Biotherapy of Zhejiang Province, Hangzhou, Zhejiang China; 6 Key Laboratory of Cancer Prevention and Intervention, China National Ministry of Education, Hangzhou, China

**Keywords:** Hepatocellular carcinoma, Hepatitis B virus, microRNA, Regulatory network

## Abstract

**Background:**

MicroRNAs (miRNAs) exhibit essential regulatory functions related to cell growth, apoptosis, development and differentiation. Dysregulated expression of miRNAs is associated with a wide variety of human diseases. As such miRNA signatures are valuable as biomarkers for disease and for making treatment decisions. Hepatitis B virus (HBV) is a major risk factor for hepatocellular carcinoma (HCC). Here we screened for miRNAs in chronic HBV associated HCC.

**Methods:**

To determine the miRNAs in HCC occurrence associated with HBV infection, we analyzed global miRNA expression profiles in 12 pairs of HCC and adjacent matched non-HCC tissues from HBV-positive and HBV-negative patients using microarray analyses. The microarray result was validated by real-time PCR in 32 HBV-positive and 24 HBV-negative patient HCC samples. The potential candidate target genes of the miRNAs were predicted by miRWalk software. Genes simultaneously predicted as targets by two or more miRNAs were subjected to GO and KEGG pathway analysis. The miRNA regulatory network analysis was performed using the Ingenuity Pathway Analysis (IPA) software.

**Results:**

Eight miRNAs (miR-223, miR-98, miR-15b, miR-199a-5p, miR-19b, miR-22, miR-451, and miR-101) were involved in HBV-unrelated HCC, 5 miRNAs (miR-98, miR-375, miR-335, miR-199a-5p, and miR-22) were involved in HBV infection, and 7 miRNAs (miR-150, miR-342-3p, miR-663, miR-20b, miR-92a-3p, miR-376c-3p and miR-92b) were specifically altered in HBV-related HCC. Gene Ontology and KEGG analyses predict that these HBV-related HCC miRNAs are involved in the regulation of: transcription, RNA polymerase II promoter, phosphorylation of proteins through MAPK signaling pathway, focal adhesion, and actin cytoskeleton. IPA analysis also suggest that these miRNAs act on AGO2, TP53, CCND1, and 11 other genes that significantly influence HCC occurrence and HBV infection.

**Conclusion:**

Our data indicates that the unique 7 miRNAs expression signature could be involved in the development HBV- related HCC.

**Electronic supplementary material:**

The online version of this article (10.1186/s12885-017-3816-1) contains supplementary material, which is available to authorized users.

## Background

Hepatocellular carcinoma (HCC) is among the most common of solid cancers with the third highest mortality worldwide [1]. Chronic hepatitis B virus (HBV) infection is a major risk factor for HCC [2]. Studies in literature indicate that several HBV-coded proteins promote malignant transformation in hepatocytes [3, 4]. HBV-related HCC has poor clinical recovery because a curative treatment is still lacking and the high rate of recurrence after treatment [5]. An understanding of the pathogenesis of HBV-associated HCC will provide insights for developing effective therapeutic and/or preventive strategies to combat this highly malignant form of cancer [6].

MicroRNAs (miRNA) constitute a recently discovered class of non-coding RNAs and are known to function in the regulation of gene expression [7, 8]. These molecules regulate the expression of as much as 30% of all mammalian protein-encoding genes. In addition to their important roles in healthy individuals, many studies have revealed that various miRNAs are involved in human carcinogenesis and other diseases. Consequently, miRNAs are being evaluated as candidates for diagnostic/prognostic biomarkers and predictors of drug response. The abnormal expression of miRNAs through transcriptional/post-transcriptional regulation or imperfect pairing with target messenger RNAs (mRNAs) of genes have been observed in disease processes [9–12]. Several studies have shown that expression of miRNAs is dysregulated in HCC compared to non-tumor liver tissues [13]. For example, miR-122 is involved in liver development, differentiation, homeostasis and metabolic functions. MiR-122 targets CUTL1 and CCNG1, and loss of miR-122 results in blocked differentiation, genomic instability, and inflammation associated with liver disease and HCC [14]. MiR-199 targets hepatocyte growth factor receptor, mammalian target of rapamycin (mTOR), and hypoxia-inducible factor (HIF1α), thereby regulating receptor tyrosine kinase and mTOR activation [15]. MiR-21 targets programmed cell death protein 4 (PDCD4) and phosphatase and tensin homolog (PTEN), thereby modulating apoptosis resistance [16]. There is a paucity of information on miRNAs engaged in HBV-related HCC and the regulatory mechanisms of these miRNAs remain largely unknown.

The present study was undertaken to investigate the expression pattern and possible function of miRNAs to provide insights on molecular mechanisms of HBV-related HCC. Results of this study can be used to provide potential candidate biomarkers for HBV-related HCC detection. By miRNA expression profile, we found that miR-150, miR-342-3p, miR-663, miR-20b, miR-92a-3p, miR-376c-3p, and miR-92b are specifically altered in HBV-related HCC. Gene Ontology (GO) and KEGG analysis suggest that these miRNAs may be involved in transcription regulation, MAPK dependent protein phosphorylation, as well as modulation of focal adhesion and actin cytoskeleton. IPA analysis also suggests that these miRNAs act on AGO2, TP53, CCND1, and 11 other genes that are implicated in the occurrence of HCC and HBV infection.

## Methods

### Patients and samples

Tumor and paired adjacent non-tumor tissue samples were obtained from 56 liver cancer patients undergoing primary tumor resection at the Sir Run Run Shaw Hospital of Zhejiang University from February 2011 to May 2015. Patients who received pre-operative chemotherapy were excluded. Among the 56 patients, 32 patients were HBV-positive, and 24 patients were HBV-negative. This study was performed in strict accordance with the recommendations from the Guide for Clinical Research provided by Sir Run Run Shaw Hospital, Zhejiang University. The protocol was approved and monitored by the Ethics Committee of Sir Run Run Shaw Hospital, Zhejiang University. Signed informed consent was obtained from each patient. The biopsies were snap-frozen in liquid nitrogen and stored at −80 °C until use.

### Microarray analysis

Microarray assay was started with 2 to 5 μg total RNA sample and performed using a service provider (LC Sciences, http://lcsciences.com/documents/microrna_faqs.pdf). Data were analyzed by first subtracting the background and then normalizing the signals using a LOWESS filter (Locally-weighted Regression). For two color experiments, the ratio of the two sets of detected signals (log2 transformed, balanced) and *p*-values of the t-test were calculated; differentially detected signals were those with less than 0.01 *p*-values.

### Real time PCR

Total RNA was purified with TRIzol reagent (Invitrogen) and reverse transcribed using a reverse transcription system (Promega, Madison, WI, USA), according to the manufacturer’s instructions. After polyadenylation, reverse transcription was performed in a 20 μl reaction volume. The reaction was incubated at 42 °C for 15 min, and then terminated by heating at 95 °C for 5 min. Real-time PCR was performed using FastStart Universal SYBR Green Master (Roche Diagnostics, Rotkreuz, Switzerland), and results analyzed with Eppendorf Real-Time Detection System (Eppendorf, Hauppauge, NY). The primer pairs used for miRNAs are shown in Additional file 1: Table S3. PCR amplification parameters were as follows: 95 °C for 5 min, followed by 40 cycles with each cycle at 95 °C for 15 s, 60 °C for 30 s and 72 °C for 30 s. Relative expression levels were calculated using the formula 2^-(CTgapdh—CTgene)^ [17]. The miRNA levels were normalized by actin housekeeping gene.

### Cluster analysis

Unsupervised hierarchical clustering was carried out using Cluster 3.0 according to methods described previously [18, 19]. Heat maps were generated in Java Treeview, the relative expression of each gene was described as the log_10_(ratio).

### Analysis of differentially expressed miRNAs

Differentially expressed miRNAs were analyzed using the significance analysis of microarrays (SAM) program according to methods described previously [18]. The target genes of selected miRNAs were predicted and analyzed by mirWalk, GO and pathway analysis.

## Results

### HBV-infection induced global changes in miRNA expression

To evaluate the effect of HBV infection on the change in expression of miRNAs, 12 pairs of samples from HCC and non-tumor tissues (including 6 HBV-positive HCC and 6 HBV-negative HCC and their non-tumor tissues) were collected. The extracted RNAs were evaluated to detect the expression of miRNAs. Using ANOVA to screen the differential expression of miRNAs at *P*-value ≤ 0.01, fold change ≥ 2 or ≤ 0.5, 225 miRNAs were detected (Additional file 1: Figure S1). This finding suggests that HBV infection may indeed affect the expression of miRNAs in liver cells, and the changes in miRNA expression may result in specific inflammation and tumorigenesis.

**Fig. 1:**
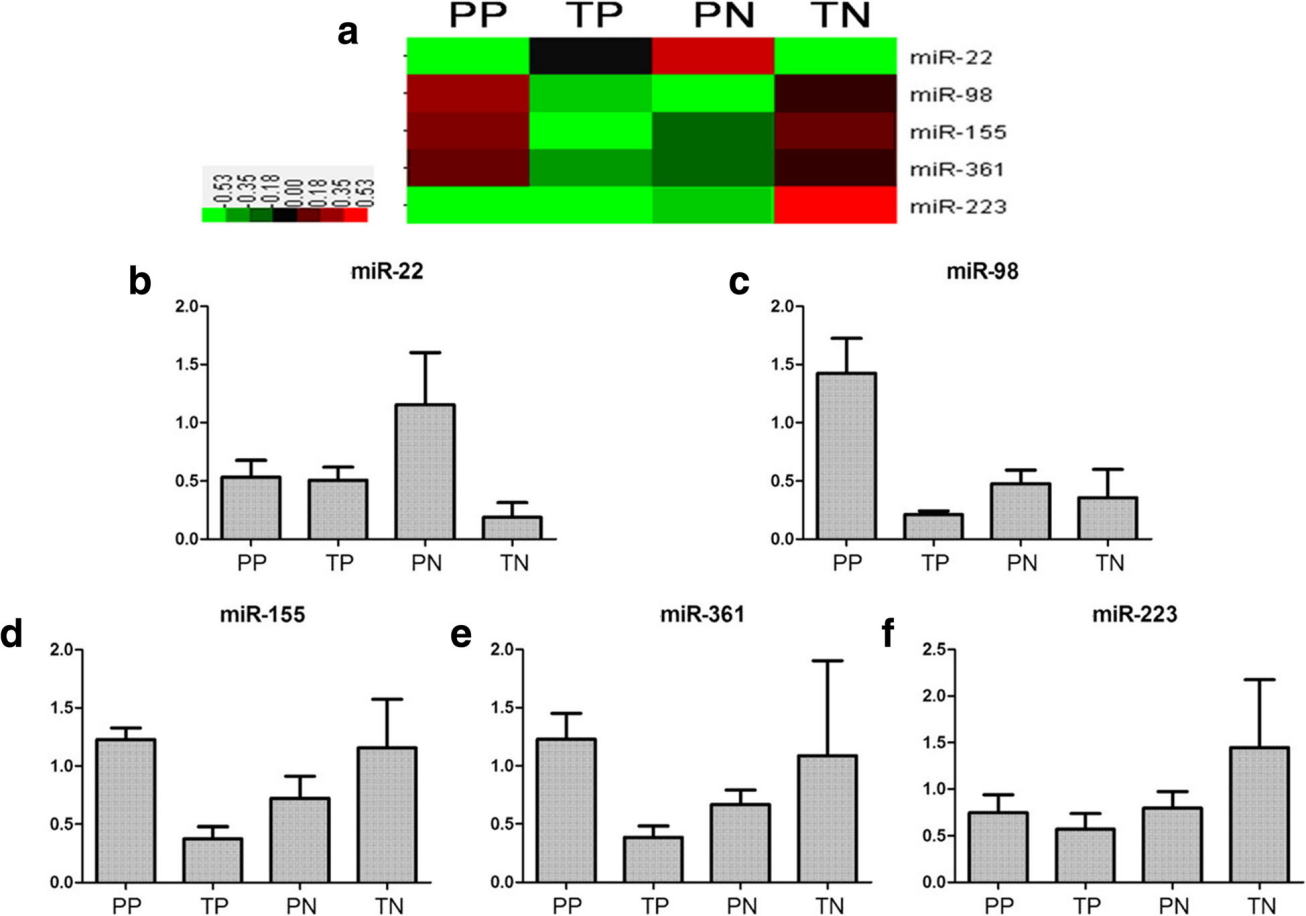
qPCR results of candidate miRNA confirm microarray data. **a** 5 miRNAs were selected for confirmation by qPCR. in HCC and ajdjacent non tumor tissues. **b** The expression of miR-22. **c** The expression of miR-98. **d** The expression of miR-155. **e** The expression of miR-361. **f** The expression of miR-223. PP = adjacent non tumor tissues of HBV-positive patients, TP = tumors of HBV-positive patients, PN = adjacent non-tumor tissues of HBV-negative patient, TN = tumors of HBV-negative patients

To examine the reliability of the array data, we selected 5 miRNAs to confirm their expression in HCC or non-tumor tissues from HBV-positive and HBV-negative patients by using qPCR. The results of qPCR were consistent with microarray data (Fig. 1).

### miRNAs are involved in the HBV infection

To determine whether miRNAs are involved in HBV infection, we compared the expressions of miRNAs between HBV-positive and HBV-negative non-tumor tissues. T-test analysis showed that 10 miRNAs were up-regulated and 15 miRNAs were down-regulated in HBV-positive non-tumor tissues compared with those in HBV-negative non-tumor tissues (Additional file 1: Table S1), thereby suggesting the possible role of these miRNAs in HBV infection. MiRNAs selected at fold change > 5 are noted as effective. Five miRNAs (miR-98, miR-375, miR-335, miR-199a-5p, and miR-22) matched the criterion.

**Fig. 2:**
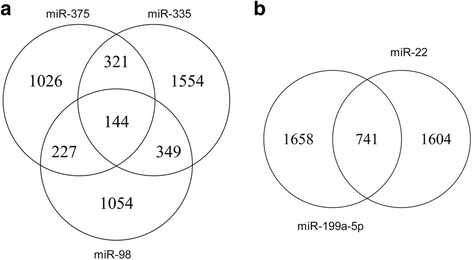
Venn diagram analysis of the relationships among target genes predicted by differentially expressed miRNAs in HBV infection. **a** Relationships among the target genes predicted by miR-98, miR-375, and miR-335. **b** Relationships among target genes predicted by miR-199a-5p and miR-22

**Fig. 3:**
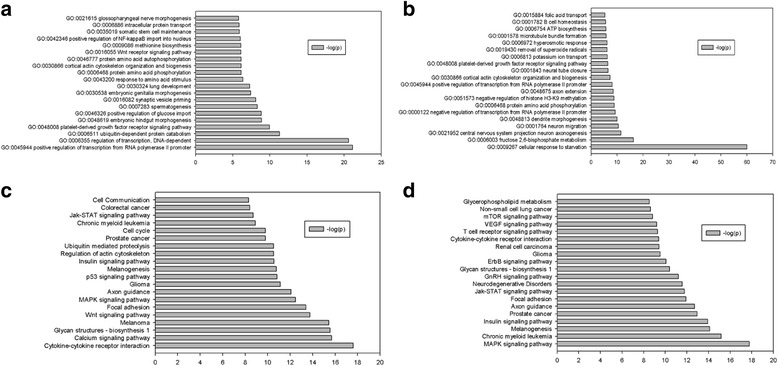
GO and pathway analysis results of the target genes predicted by differentially expressed miRNAs in HBV infection. -log10(P) indicates the GO score related to genes with the biological process by *P*-value. **a** Main biological processes influenced by genes targeted by two or more miRNAs (miR-98, mir-375, and miR-335). **b** Main biological processes influenced by genes targeted by miR-199a-5p, and miR-22. **c** Main pathway influenced by genes targeted by two or more miRNAs from miR-98, mir-375, and miR-335. **d** Main pathway influenced by genes targeted by miR-199a-5p, and miR-22

The process of selecting predicted target genes that will undergo GO and KEGG pathway analysis was performed as previously described (Fig. 2). GO analysis showed that the most significant biological processes for miR-98, miR-375, and miR-335 include positive regulation of transcription from RNA polymerase II promoter, regulation of transcription, DNA-dependent and ubiquitin-dependent protein catabolism, platelet-derived growth factor receptor signaling pathway, embryonic hindgut morphogenesis (Fig. 3a). In the same way, the processes targeted by miR-199a-5p and miR-22 were cellular response to starvation, fructose 2, 6-bisphosphate metabolism, central nervous system projection neuron axon genesis, neuron migration, and dendrite morphogenesis (Fig. 3b).

KEGG pathway analysis showed that the predicted target genes related to miR-98, miR-375, and miR-335 were involved in cytokine-cytokine receptor interaction, calcium signaling pathway, glycan structures - biosynthesis 1, melanoma, and Wnt signaling pathway (Fig. 3c), whereas the predicted target genes of miR-199a-5p and miR-22 were related to MAPK signaling pathway, chronic myeloid leukemia, melanogenesis, insulin signaling pathway, and prostate cancer (Fig. 3d).

### HBV-infection alters the expression of miRNAs specifically involved in the carcinogenesis of HCC

To determine which miRNAs specifically function in HBV-induced HCC, we first hypothesized that these pivotal miRNAs should be up-regulated or down-regulated after HBV infection and continually function during carcinogenesis. Hence, miRNAs that were either up-regulated or down-regulated after HBV infection were determined by comparing HBV-negative non-tumor tissues and HBV-positive non-tumor tissues (Additional file 1: Table S1). MiRNAs that were up- or down-regulated at fold change >2 in HBV-positive HCC tissues were selected by comparing HBV-positive non-tumor tissues and HBV-positive tumors (Table 1). After compared the expression of miRNAs in HBV-negative non-tumor tissues, HBV-positive non-tumor tissues, HBV-negative tumor tissues and HBV-positive tumor tissues, we selected 12 miRNAs exhibited consistently altered expression levels during HBV infection to HBV-related HCC. Among these 12 miRNAs, 5 were consistently up-regulated and 7 were consistently down-regulated. The identity of the 12 miRNAs is as follows: miR-21, miR-20b, miR-92a-3p, miR-92b, miR-376c-3p, miR-150, miR-451, miR-101, miR-424, miR-342-3p, miR-122a, and miR-663. Results demonstrated that these 12 miRNAs played a regulatory role in the occurrence of HCC caused by HBV infection. By comparing HBV-negative non-tumor tissues and HBV-negative tumors, we found that the expressions of 11 miRNAs increased, whereas the expressions of 15 miRNAs decreased (Additional file 1: Table S2). To obtain miRNAs that are specific to HBV-induced HCC, we deduct the same miRNAs that function in HBV-unrelated HCC. Finally, we found 7 HBV-related miRNAs that are involved in HCC occurrence after removing 5 non-specific miRNAs (miR-21, miR-451, miR-101, miR-424, and miR-122a). Ultimately, miR-20b, miR-92a-3p, miR-92b, miR-376c-3p, miR-150, miR-342-3p, and miR-663 were selected. These miRNAs, 4 up-regulated and 3 down-regulated, are specifically involved in HBV-induced HCC. Genes that are targeted by the 7 selected miRNAs may be involved in critical functional pathways that lead to HBV-related HCC.

**Table 1:** Differentially expressed microRNAs in HCC versus adjacent non-tumor tissues from HBV-positive patients

		HBV + tumor		HBV + non-tumor		
Reporter name	*p*-value	Mean	StDev	Mean	StDev	Fold change
miR-368	1.01E-12	3188	99	109	7	29.17
miR-487b	5.33E-15	2983	183	106	6	28.17
miR-127	6.02E-09	5319	145	218	20	24.36
miR-19b	1.33E-15	18,147	452	2382	83	7.62
miR-22	5.79E-08	3234	51	574	47	5.64
miR-181a	1.86E-09	5512	209	1519	105	3.63
miR-25	7.89E-10	8586	512	3491	201	2.46
miR-191	1.54E-10	8562	267	3521	155	2.43
miR-125b	1.86E-10	13,712	365	5849	270	2.34
miR-125a	1.04E-08	5357	389	2408	161	2.22
miR-27a	3.79E-11	12,665	409	5814	233	2.18
miR-24	1.03E-05	10,905	996	5138	161	2.12
miR-143	8.95E-10	8383	426	4040	186	2.08
miR-638	1.44E-10	22,023	780	10,794	454	2.04
miR-103	1.05E-09	7394	303	3661	199	2.02
miR-15a	6.98E-09	2089	79	4434	275	−2.13
miR-195	3.82E-10	7697	323	17,122	921	−2.22
miR-146a	1.36E-09	4030	253	10,074	647	−2.50
miR-361	3.78E-09	1933	156	6058	349	−3.13
miR-101	2.44E-09	964	97	3290	267	−3.45
miR-671	6.21E-11	1481	93	6074	245	−4.17
miR-152	5.71E-10	1118	42	4799	315	−4.35
miR-146b	1.13E-10	2357	230	11,701	826	−5.00
miR-451	3.67E-11	1985	90	10,252	700	−5.26
miR-98	2.16E-08	1009	180	5705	612	−5.56
miR-375	1.11E-08	623	61	4157	160	−6.67
miR-768-3p	4.00E-15	837	27	7214	281	−8.33
miR-10a	2.96E-11	351	41	4116	410	−11.11
miR-768-5p	2.36E-11	611	64	9400	606	−14.29
miR-335	1.57E-07	261	72	4499	692	−16.67
miR-29c	2.08E-10	416	67	7547	806	−16.67
miR-155	6.00E-12	310	19	5898	589	−20.00

### Functional analysis of miRNAs in HBV-induced HCC

**Fig. 4:**
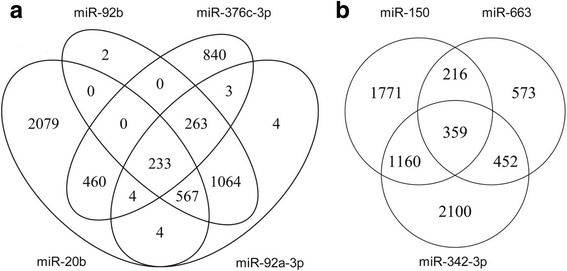
Venn diagram analysis of the relationship among target genes predicted by differentially expressed miRNAs which are involved in HBV-induced HCC. **a** Relationship among target genes predicted by miR-20b, miR-92a-3p, miR-92b, and miR-376c-3p. **b** Relationship among target genes predicted by miR-150, miR-342-3p, and miR-663

**Fig. 5:**
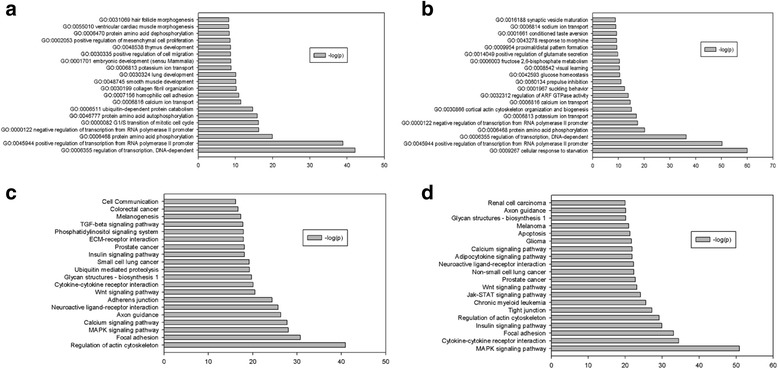
GO and pathway analysis result of the target genes predicted by differentially expressed miRNAs which are involved in HBV-induced HCC. -log10(P) indicates the GO score related to genes in the biological process by *P*-value. **a** Main biological processes influenced by genes targeted by two or more miRNAs from miR-20b, miR-92a-3p, miR-92b, and miR-376c-3p. **b** Main biological processes influenced by genes targeted by two or more miRNAs from miR-150, miR-342-3p, and miR-663. **c** Main pathways influenced by genes targeted by two or more miRNAs from miR-20b, miR-92a-3p, miR-92b, and miR-376c-3p. **d** Main pathways influenced by genes targeted by two or more miRNAs from miR-19b, miR-101, and miR-199a-5p

The functions of the 7 miRNAs with possible specific involvement in HBV-induced HCC were extensively analyzed. miRWalk software was used to predict the target genes of these miRNAs. Genes predicted simultaneously as target genes by two or more miRNAs were selected and subjected to GO and KEGG pathway analysis as previously described (Fig. 4). Results showed that the most significant biological processes targeted by at least two of various miRNAs (miR-20b, miR-92a-3p, miR-92b, and miR-376c-3p) were regulation of transcription, DNA-dependent positive regulation of transcription from RNA polymerase II promoter, protein amino acid phosphorylation, negative regulation of transcription from RNA polymerase II promoter, and G1/S transition of mitotic cell cycle (Fig. 5a). Similarly, the processes targeted by at least two of several miRNAs (miR-150, miR-342-3p, and miR-663) were cellular response to starvation, positive regulation of transcription from RNA polymerase II promoter, regulation of transcription, DNA-dependent protein amino acid phosphorylation, and negative regulation of transcription from RNA polymerase II promoter (Fig. 5b). The up-regulated and down-regulated miRNAs (miR-20b, miR-92a-3p, miR-92b, miR-376c-3p, miR-150, miR-342-3p, and miR-663) may be involved in the regulation of transcription, DNA-dependent positive regulation of transcription from RNA polymerase II promoter, protein amino acid phosphorylation, and negative regulation of transcription from RNA polymerase II promoter.

Pathway analysis showed that the predicted target genes related to miR-20b, miR-92a-3p, miR-92b, and miR-376c-3p were involved in regulation of actin cytoskeleton, focal adhesion, MAPK signaling pathway, calcium signaling pathway, and axon guidance (Fig. 5c). The predicted target genes of miR-150, miR-342-3p, and miR-663 were related to MAPK signaling pathway, cytokine-cytokine receptor interaction, focal adhesion, insulin signaling pathway, and regulation of actin cytoskeleton (Fig. 5d). These collective findings suggest that miRNAs may serve functions in HBV-induced HCC through MAPK signaling pathway, focal adhesion, and regulation of actin cytoskeleton.

**Fig. 6:**
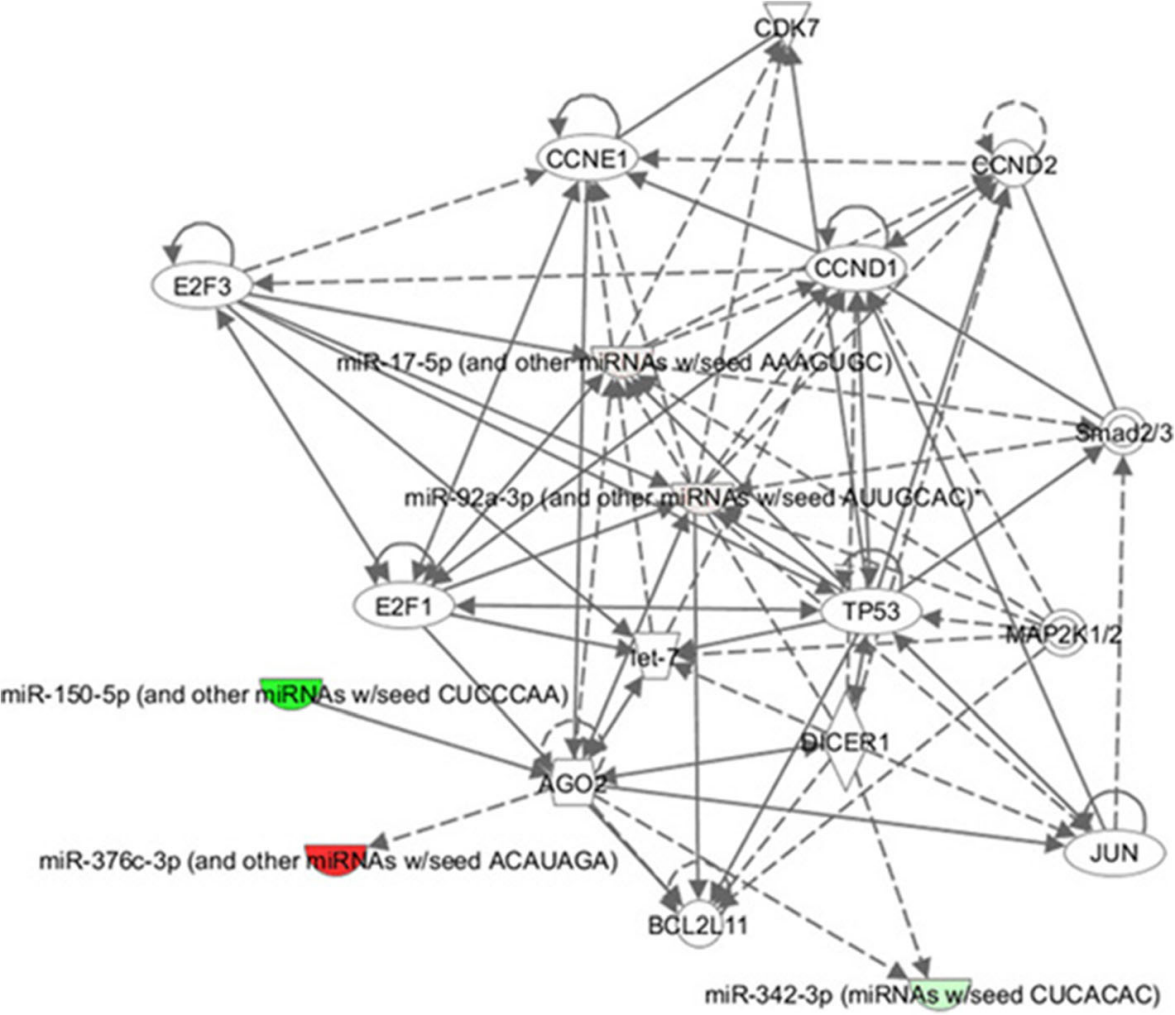
Results of IPA analysis of the target genes predicted by differentially expressed miRNAs involved in HBV-indcued HCC. IPA-obtained network showing the relationships among 6 co-regulated miRNAs (miR-150, miR-342-3p, miR-92a-3p, miR-92b, and miR-376c-3p)

The ingenuity pathway analysis (IPA) is able to identify published direct binding partners, transcriptional regulators, and translational regulators of specific molecules. The functional pathways regulated by all the selected miRNAs can manifest the co-regulated relationships of these miRNAs. In our study, a network that includes 6 of 7 selected miRNAs (miR-150, miR-342-3p, miR-20b, miR-92, miR-368, and miR-92b) was shown based on accepted databases of molecular interactions reported in the literature using IPA (Fig. 6). These 6 miRNAs act on AGO2, TP53, CCND1, and other 11 genes that play important roles in HCC occurrence and HBV infection.

## Discussion

HBV infection is a major health problem that leads to a significant rise in mortality and is reported to be closely related to HCC [20]. HBV contains four open reading frames (ORFs), namely, S, P, X, and pre C, whose products are HBeAg, HBcAg, HBsAg, and HBx, respectively [21]. Expression of HBV proteins was previously demonstrated to modulate the expression of some genes that likely contribute to the pathogenesis of HCC [3, 4]. Especially HBx plays a critical role in HBV-related HCC [21]. HBx can stop cell death mediated by p53, Fas, and transforming growth factor-β [22, 23], thereby implying the importance of regulation of apoptosis in the occurrence of HBV-related HCC. One report has shown that down-regulating the expressions of HBsAg, HBcAg, p21, and Rb proteins in HCC increases the propensity for HCC occurrence, indicating that these proteins are tumor suppressors and would influence the course of cell cycle and apoptosis [24]. All of the above information implied that HBV infection could affect cell cycle and survival and could eventually lead to HCC.

In our IPA results, the 6 selected miRNAs (miR-150, miR-342-3p, miR-20b, miR-92a-3p, miR-92b, and miR-376c-3p) are shown to comprise a network which linked themselves among AGO2, DICER1, BCL2L11, CCND1, CCND2, CCNE1, CDK7, E2F1, E2F3, TP53, and four other genes (Fig. 6). Argonaute RISC catalytic component 2 (AGO2) and dicer 1 (DICER1) were highlighted. AGO2 plays a role for RNA interference in regulating the chromatin structure. AGO2 may interact with dicer1 and play a role in short-interfering-RNA-mediated gene silencing. Previous reports have indicated that HBsAg and HBcAg co-localized with AGO2. Moreover, HBV-specific miRNAs, together with AGO2, play a role in the viral life cycle [25]. So far, no HBV-encoded miRNA has been identified [26]. Other genes shown in the IPA network were involved in regulation of cellular regulatory pathway. Thus, we can speculate that cellular miRNAs may function in HBV-induced HCC either by targeting cellular transcriptions factors required for HCC occurrence or by directly binding to HBV transcripts to affect HBV gene expression.

Many functional pathways are reportedly regulated during HBV infection. The deregulation of signaling pathways includes MAPKs, p53, Wnt/β-catenin, transforming growth factor β (TGF β), cytokines, and Jak-STAT [27, 28], and results to down-regulation of tumor suppressor gene expression and/or up-regulation of oncogene expression [29]. Coincidently, the pathway analysis results showed that the 7 miRNAs selected through the process were closely involved in MAPK signaling pathway, TGF-beta signaling pathway, cytokine-cytokine receptor interaction, wnt signaling pathway, Jak-STAT signaling pathway, and apoptosis (Fig. 5c, d). Thus, HBV infection may induce HCC mainly by acting on these miRNAs to influence liver cell states.

Products of HBV contribute to HBV-associated HCC; some of these can prompt cell cycle regulatory pathways [30]. For example, studies demonstrated that HBx, a product of HBV, triggered the occurrence of HCC by decreasing the levels of cell cycle proteins that prevent progression into G1 phase and increasing the levels of cell cycle proteins active in the G1 phase [31]. Cell cycle regulation plays an important role in HBV-induced HCC. In our study, genes of cell cycle proteins, namely, cyclin D1 (CCND1), cyclin D2 (CCND2), cyclin E1 (CCNE1), cyclin-dependent kinase 7 (CDK7), transcription factor 1 (E2F1), transcription factor 3 (E2F3), and tumor protein p53 (TP53) appeared in the IPA network (Fig. 6). The proteins encoded by these genes are obviously involved in cell cycle. Cyclin D1, cyclin D2 and cyclin E1 belong to the highly conserved cyclin family. Among these proteins, cyclin D1 and cyclin D2 form a complex with and function as a regulatory subunit of CDK4 or CDK6, whose activity is required for cell cycle G1/S transition. In addition, cyclin E1 functions with CDK2, whose activity is also necessary in G1/S transition [32]. Thus, mutations, amplification, and overexpression of these genes are frequently observed in HBV-related HCC [33]. Cyclin-dependent kinase 7 serves as a direct link between the regulation of transcription and the cell cycle [34]. E2F1 and E2F3 are members of the E2F family, their target genes function in DNA replication and repair, cell cycle regulation, cell cycle checkpoint, cell death, differentiation. E2F transcription factors are key targets of the retinoblastoma (Rb) tumor suppressor [35]. Previous research indicated that P53 was the most frequently altered pathway in HBV-related HCC, and TP53 was associated with shorter survival only in HBV-related HCC [36]. Coincidently, our target gene analysis showed that all the 4 up-regulated miRNAs (miR-20b, miR-92a-3p, miR-92b, and miR-376c-3p) target TP53, cyclin-dependent kinase inhibitor 1A (CDKN1A) and cyclin-dependent kinase inhibitor 2A (CDKN2A). Our study verified that the expression of TP53 and CDKN1A were decreased in HBV positive tumors compared with HBV positive non tumor tissues (Additional file 1: Figure S2). TP53 is a classical suppressor gene that is related to cell cycle and accumulation of genetic changes. The proteins encoded by CDKN1A and CDKN2A can inhibit the activity of cyclin-CDK and function as tumor suppressors to control cell cycle in HBV-related HCC [37, 38]. In addition, all down-regulated miRNAs (miR-150, miR-342-3p, and miR-663) target baculoviral IAP repeat containing 5 (BIRC5), CCND1, and protein tyrosine kinase 2 (PTK2). The expression of these three genes was increased in HBV positive tumors compared with HBV positive non-tumor tissues (Additional file 1: Figure S2). The product of PTK2 acts on cell cycle by regulating the tumor suppressor p53 [39]. Based on these results, aberrant regulation of cell cycle may be partially attributed to the occurrence of HBV-associated HCC.

In previous studies, a strong connection between apoptosis and HBV-related HCC has been shown [40, 41]. IPA result showed that the 6 co-regulated miRNAs function on TP53, E2F1, E2F3, and BCL2L11 directly or indirectly (Fig. 6). Thus, it is reasonable to speculate that these miRNAs play a role in occurrence of HCC through HBV infection regulating apoptosis. TP53 in the regulation of apoptosis was reportedly essential in HBV-induced HCC [42]. E2F1 and E2F3 can mediate cell proliferation and p53-dependent/independent apoptosis by binding to protein pRB [43, 44]. The protein encoded by BCL2-like 11 (BCL2L11) belongs to the BCL-2 protein family and acts as an apoptotic activator that serves a function in HCC occurrence [45]. Our study showed that high expression of miR-20b, miR-92a-3p, and miR-92b can down-regulate Fas-associated protein with death domain (FADD), which is known as an adaptor molecule that bridges the Fas-receptor [46] and is involved in apoptosis [47]. Moreover, the up-regulated miRNAs (miR-20b, miR-92a-3p, miR-92b, and miR-376c-3p) target BH3-interacting domain (BID), TP53 and PTEN. Consistently, the expression of gene ERK, TP53 and PTEN were decreased in HBV positive tumors compared with HBV positive non-tumor tissues (Additional file 1: Figure S2A). All down-regulated miRNAs (miR-150, miR-342-3p, and miR-663) target B-cell lymphoma/leukemia 2 (BCL2), BIRC5 and PTK2. The expression of these three genes was increased in HBV positive tumors compared with HBV positive non-tumor tissues (Additional file 1: Figure S2B). These genes were reportedly closely involved in apoptosis regulation in HBV-related HCC [37, 39, 47–52]. Moreover, lower expression of miR-150 and miR-342-3p up-regulated Bcl-2–associated X protein and CASP8 and FADD-like apoptosis regulator (CFLAR), which have apoptotic activities [49, 53]. All these outcomes imply the importance of apoptosis regulation by miRNAs in HBV-related HCC.

From aforementioned results, we can speculate that a co-regulation exists among the 7 selected miRNAs, thereby promoting HBV-related HCC. First, 6 selected miRNAs comprised a network; these miRNAs linked themselves among AGO2, DICER1, BCL2L11, CCND1, CCND2, CCNE1, CDK7, E2F1, E2F3, TP53, and four other genes. AGO2 and DICER2, together with miRNAs and HBV products, affect viral life cycle. Other genes, however, mainly function in cellular regulatory pathways. So far, no HBV-encoded miRNA has been identified. Thus, we speculate that cellular miRNAs may function in HBV-induced HCC at the transcription level either by targeting cellular transcription factors required for HCC occurrence or by a directly binding to HBV transcripts to affect HBV gene expression. Furthermore, we found that all 7 HBV-specific miRNAs participate in cell cycle and apoptosis by regulating various genes that code for cell cycle and apoptosis regulators. All these miRNAs act together to regulate a variety of physiological functions, which ultimately lead to HCC. We may use these 7 miRNAs as possible biomarkers that can be applied to HBV-induced HCC detection, early intervention, and recurrence. However, all of these predictions are a good starting point for the involvement of miRNAs in HBV-related HCC, and the exact functions of the miRNAs identified in this article need experimental verification.

## Conclusions

Aberrations in miRNA expression are correlated to cancer progression. In this study, we analyzed global miRNA expression profiles in HCC and adjacent matched non-HCC tissues from HBV-positive and HBV-negative patients. Our data indicates that the unique 7 miRNAs (miR-150, miR-342-3p, miR-663, miR-20b, miR-92a-3p, miR-376c-3p and miR-92b) expression signature could be involved in the development of HBV- related HCC, suggesting interesting potential novel therapeutic options. However, further functional studies are needed to clarify the role of these miRNAs in HBV-related HCC.

## Additional file

Additional file 1: Figure S1. The expression profiles of global miRNAs in HCC and adjacent non-tumor tissues of HBV-positive and HBV-negative patients. A heatmap of global miRNAs in HCC and non-tumor tissues obtained from 12 people (6 tumor tissues and 6 adjacent non-tumor tissues from 6 HBV-positive HCC patients; 6 tumor tissues and 6 adjacent non-tumor tissues from 6 HBV-negative HCC patients). TN = tumors of HBV- negative patients, PN = adjacent non-tumor tissues of HBV-negative patient, TP = tumors of HBV-positive patients, PP = adjacent non-tumor tissues of HBV-positive patients. **Figure S2.** Expression of partial target genes predicted by differentially expressed miRNAs. (**A**) Expression of gene TP53, CDKN1A, ERK and PTEN in 32 pairs of HBV positive adjacent non-tumor and tumor tissues. (**B**) Expression of gene BIRC5, CCND1, PTK2 and BCL2 in 32 pairs of HBV positive adjacent non-tumor and tumor tissues. ***P* < 0.01, ****P* < 0.001, *****P* < 0.0001. **Table S1.** Differentially expressed microRNAs in HCC versus normal tissues from HBV-negative patients. **Table S2.** Differentially expressed microRNAs in normal tissues from HBV-positive versus HBV-negative patients. **Table S3.** Primers used in real time PCRs for detecting microRNAs expression. (DOC 610 kb)

## Abbreviation

AGO2: Argonaute RISC catalytic component 2
BCL2: B-cell lymphoma/leukemia 2
BCL2L11: BCL2-like 11
BID: BH3-interacting domain
BIRC5: Baculoviral IAP repeat containing 5
CCND1: Cyclin D1
CCND2: Cyclin D2
CCNE1: Cyclin E1
CDK7: Cyclin-dependent kinase 7
CDKN1A: Cyclin-dependent kinase inhibitor 1A
CDKN2A: Cyclin-dependent kinase inhibitor 2A
CFLAR: CASP8 and FADD-like apoptosis regulator
E2F1: Transcription factor 1
E2F3: Transcription factor 3
FADD: Fas-associated protein with death domain
GO: Gene Ontology
HBV: Hepatitis B virus
HCC: Hepatocellular carcinoma
HIF1α: Hypoxia-inducible factor
IPA: Ingenuity Pathway Analysis
miRNA: microRNA
mRNAs: messenger RNAs
mTOR: mammalian target of rapamycin
ORFs: Open reading frames
PDCD4: Programmed cell death protein 4
PTEN: Phosphatase and tensin homolog
PTK2: Protein tyrosine kinase 2
TGF β: Transforming growth factor β
TP53: Tumor protein p53
