# Supplementary material for: MicroRNA profile in HBV-induced infection and hepatocellular carcinoma
Source: BMC Cancer. 2017 Dec 1;17:805. doi: 10.1186/s12885-017-3816-1 (PMC5709924; doi:10.1186/s12885-017-3816-1)
Supplement: Additional file 1: Figure S1. — The expression profiles of global miRNAs in HCC and adjacent non-tumor tissues of HBV-positive and HBV-negative patients. A heatmap of global miRNAs in HCC and non-tumor tissues obtained from 12 people (6 tumor tissues and 6 adjacent non-tumor tissues from 6 HBV-positive HCC patients; 6 tumor tissues and 6 adjacent non-tumor tissues from 6 HBV-negative HCC patients). TN = tumors of HBV- negative patients, PN = adjacent non-tumor tissues of HBV-negative patient, TP = tumors of HBV-positive patients, PP = adjacent non-tumor tissues of HBV-positive patients. Figure S2. Expression of partial target genes predicted by differentially expressed miRNAs. (A) Expression of gene TP53, CDKN1A, ERK and PTEN in 32 pairs of HBV positive adjacent non-tumor and tumor tissues. (B) Expression of gene BIRC5, CCND1, PTK2 and BCL2 in 32 pairs of HBV positive adjacent non-tumor and tumor tissues. **P < 0.01, ***P < 0.001, ****P < 0.0001. Table S1. Differentially expressed microRNAs in HCC versus normal tissues from HBV-negative patients. Table S2. Differentially expressed microRNAs in normal tissues from HBV-positive versus HBV-negative patients. Table S3. Primers used in real time PCRs for detecting microRNAs expression. (DOC 610 kb) [file 12885_2017_3816_MOESM1_ESM.doc]

**MicroRNA Profile in HBV-Induced Infection and Hepatocellular Carcinoma**

Guanyu Wang^1*^, Fulu Dong^2*^, Zhiyao Xu^3^, Sherven Sharma^4^, Xiaotong Hu^5^, Dafang Chen^3^, Lumin Zhang^5^, Jinping Zhang^2§^, and Qinghua Dong^3,6§^

§Correspondence and requests for materials should be addressed to Q.D. ([dongqinghua@zju.edu.cn](mailto:dongqinghua@zju.edu.cn)); J.Z. ([j_pzhang@suda.edu.cn](mailto:j_pzhang@suda.edu.cn))

*These authors contributed equally to this work: Guanyu Wang & Fulu Dong


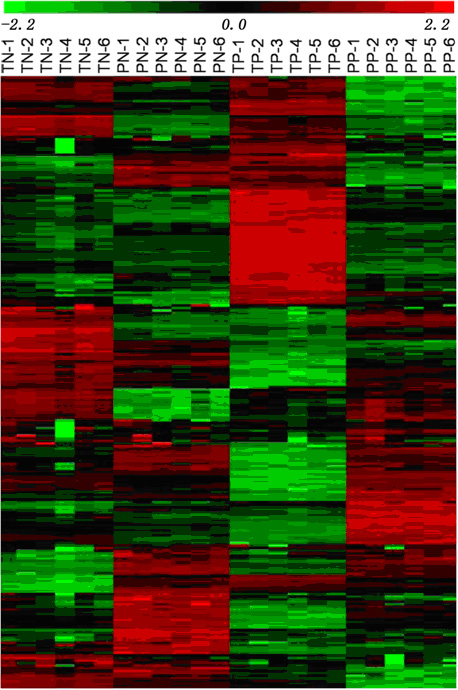


**Figure S1** The expression profiles of global miRNAs in HCC and adjacent non-tumor tissues of HBV-positive and HBV-negative patients. A heatmap of global miRNAs in HCC and non-tumor tissues obtained from 12 people (6 tumor tissues and 6 adjacent non-tumor tissues from 6 HBV-positive HCC patients; 6 tumor tissues and 6 adjacent non-tumor tissues from 6 HBV-negative HCC patients). TN = tumors of HBV- negative patients, PN = adjacent non-tumor tissues of HBV-negative patient, TP = tumors of HBV-positive patients, PP = adjacent non-tumor tissues of HBV-positive patients.


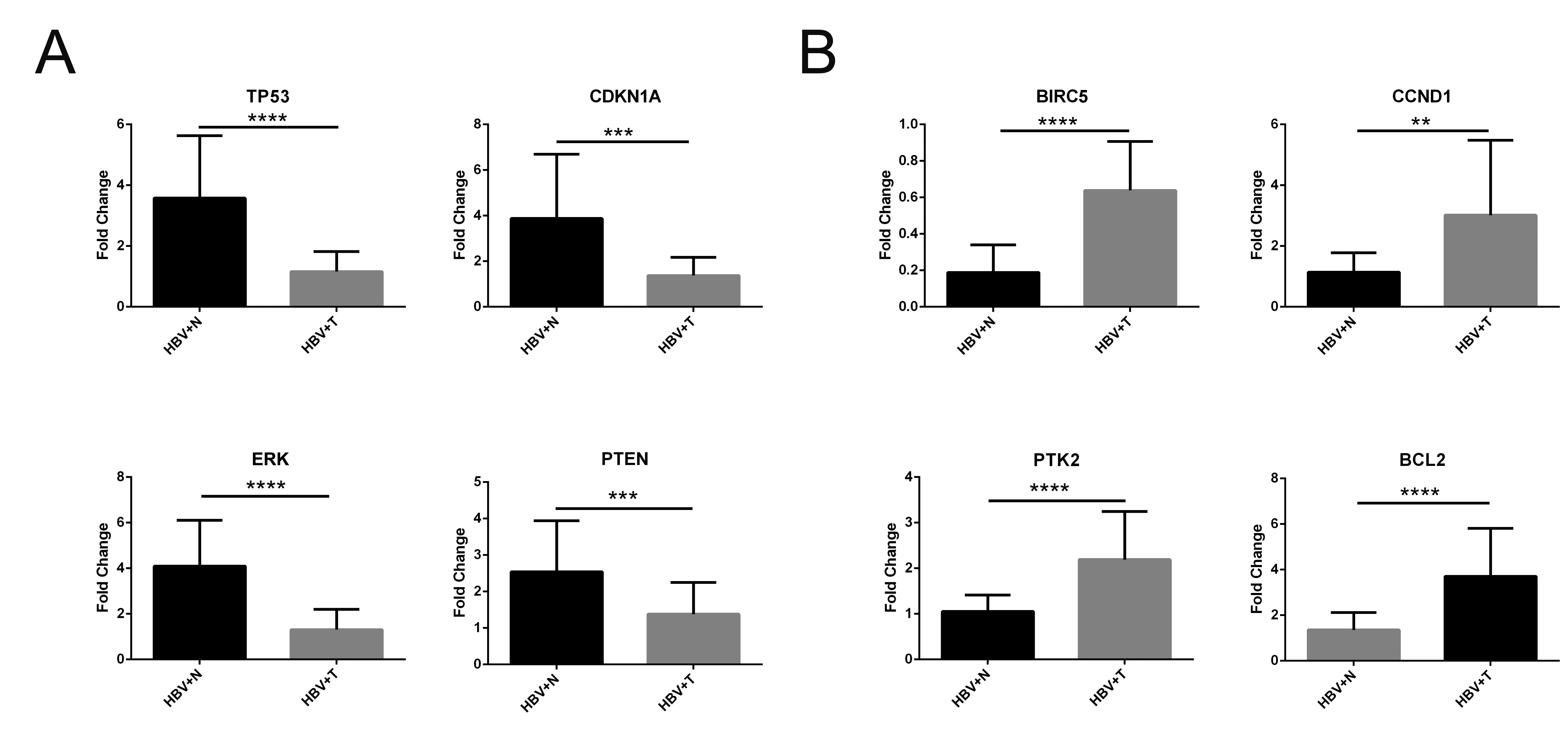


**Figure S2** Expression of partial target genes predicted by differentially expressed miRNAs. (**A**) Expression of gene TP53, CDKN1A, ERK and PTEN in 32 pairs of HBV positive adjacent non-tumor and tumor tissues. (**B**) Expression of gene BIRC5, CCND1, PTK2 and BCL2 in 32 pairs of HBV positive adjacent non-tumor and tumor tissues. ** *P*< 0.01, *** *P*< 0.001, **** *P*< 0.0001.

**Table S1** Differentially expressed miRNAs in adjacent non-tumor tissues from HBV-positive versus HBV-negative patients

|  |  | **HBV-** non-tumor | | **HBV+** non-tumor | |  |
| --- | --- | --- | --- | --- | --- | --- |
| **Reporter Name** | **p-value** | **Mean** | **StDev** | **Mean** | **StDev** | **Fold change** |
| miR-98 | 1.27E-08 | 415 | 77 | 5,705 | 612 | 13.74 |
| miR-375 | 4.31E-11 | 357 | 28 | 4,157 | 160 | 11.65 |
| miR-335 | 5.82E-10 | 411 | 59 | 4,499 | 692 | 10.93 |
| miR-155 | 1.17E-08 | 1,961 | 186 | 5,898 | 589 | 3.01 |
| miR-10a | 3.77E-07 | 1,432 | 63 | 4,116 | 410 | 2.87 |
| -miR-152 | 1.33E-09 | 1,836 | 121 | 4,799 | 315 | 2.61 |
| let-7e | 3.02E-07 | 9,368 | 1,119 | 22,394 | 2,273 | 2.39 |
| miR-361 | 1.68E-09 | 2,552 | 166 | 6,058 | 349 | 2.37 |
| miR-146a | 4.54E-09 | 4,612 | 280 | 10,074 | 647 | 2.18 |
| miR-29c | 2.64E-06 | 3,645 | 531 | 7,547 | 806 | 2.07 |
| miR-214 | 1.82E-07 | 13,356 | 1,298 | 6,586 | 475 | -2.04 |
| miR-107 | 2.99E-08 | 6,934 | 473 | 3,355 | 254 | -2.08 |
| miR-103 | 7.26E-10 | 7,728 | 333 | 3,661 | 199 | -2.13 |
| miR-99a | 2.89E-08 | 6,707 | 548 | 3,132 | 212 | -2.13 |
| miR-181a | 5.04E-09 | 3,358 | 187 | 1,519 | 105 | -2.22 |
| miR-145 | 4.19E-07 | 14,190 | 1,413 | 6,330 | 312 | -2.22 |
| miR-638 | 1.22E-07 | 24,436 | 2,047 | 10,794 | 454 | -2.27 |
| miR-451 | 2.44E-09 | 23,522 | 1,263 | 10,252 | 700 | -2.27 |
| miR-100 | 2.79E-06 | 8,751 | 480 | 3,327 | 424 | -2.63 |
| miR-125b | 1.25E-09 | 17,846 | 1,339 | 5,849 | 270 | -3.03 |
| miR-19b | 1.70E-07 | 8,042 | 848 | 2,382 | 83 | -3.33 |
| miR-125a | 5.94E-11 | 8,376 | 477 | 2,408 | 161 | -3.45 |
| miR-150 | 2.34E-09 | 5,937 | 417 | 1,422 | 144 | -4.17 |
| miR-199a-5p | 6.74E-10 | 7,720 | 588 | 1,011 | 122 | -5.88 |
| miR-22 | 1.39E-12 | 6,696 | 514 | 574 | 47 | -11.11 |

**Table S2** Differentially expressed miRNAs in HCC versus adjacent non-tumor tissues from HBV-negative patients

|  |  | **HBV-tumor** | | **HBV-** non-tumor | |  |
| --- | --- | --- | --- | --- | --- | --- |
| **Reporter Name** | **p-value** | **Mean** | **StDev** | **Mean** | **StDev** | **Fold change** |
| miR-223 | 5.94E-10 | 6,227 | 438 | 634 | 21 | 9.83 |
| miR-98 | 6.42E-09 | 3,367 | 531 | 415 | 77 | 8.11 |
| miR-15b | 3.91E-11 | 15,726 | 491 | 2,713 | 151 | 5.80 |
| miR-200b | 2.82E-09 | 11,008 | 552 | 2,310 | 213 | 4.77 |
| miR-21 | 7.27E-10 | 52,083 | 931 | 15,052 | 631 | 3.46 |
| miR-181b | 3.32E-09 | 3,621 | 235 | 1,188 | 43 | 3.05 |
| let-7i | 5.91E-09 | 15,775 | 757 | 5,820 | 485 | 2.71 |
| miR-25 | 9.61E-10 | 9,277 | 540 | 3,617 | 240 | 2.57 |
| miR-155 | 9.89E-08 | 4,892 | 540 | 1,961 | 186 | 2.49 |
| let-7e | 9.15E-07 | 23,348 | 1,508 | 9,368 | 1,119 | 2.49 |
| miR-361 | 1.36E-09 | 5,756 | 268 | 2,552 | 166 | 2.26 |
| miR-150 | 3.15E-07 | 12,647 | 392 | 5,937 | 417 | 2.13 |
| miR-148a | 1.20E-07 | 7,553 | 288 | 15,563 | 1,160 | -2.04 |
| miR-424 | 1.62E-07 | 2,032 | 123 | 4,304 | 388 | -2.13 |
| miR-192 | 1.60E-08 | 11,015 | 726 | 23,933 | 1,798 | -2.04 |
| miR-145 | 3.95E-08 | 5,461 | 341 | 14,190 | 1,413 | -2.63 |
| miR-215 | 3.03E-07 | 3,481 | 448 | 9,333 | 1,137 | -2.70 |
| miR-122a | 1.11E-09 | 24,410 | 707 | 74,052 | 4,236 | -3.03 |
| miR-29c | 1.10E-07 | 1,084 | 160 | 3,645 | 531 | -3.33 |
| miR-100 | 1.69E-11 | 2,551 | 129 | 8,751 | 480 | -3.45 |
| miR-194 | 6.17E-10 | 3,334 | 179 | 13,580 | 1,172 | -4.00 |
| miR-99a | 1.29E-10 | 1,314 | 68 | 6,707 | 548 | -5.00 |
| miR-199a-5p | 1.19E-11 | 903 | 46 | 7,720 | 588 | -8.33 |
| miR-19b | 5.88E-10 | 856 | 50 | 8,042 | 848 | -9.09 |
| miR-22 | 3.67E-12 | 650 | 29 | 6,696 | 514 | -10.0 |
| miR-451 | 4.86E-14 | 1,816 | 106 | 23,522 | 1,263 | -12.5 |
| miR-101 | 2.23E-09 | 432 | 47 | 6,362 | 296 | -14.29 |

**Table S3** Primers used in real time PCRs for detecting miRNAs expression

| **Primer Name** | **Sequence (5' to 3')** |
| --- | --- |
| miR-19b-RT | 5’CTCAACTGGTGTCGTGGAGTCGGCAATTCAGTTGAGTCAGTTTT3’ |
| miR-19b F1 | 5’ACACTCCAGCTGGGTGTGCAAATCCATGCA 3’ |
| miR-22-RT | 5’ CTCAACTGGTGTCGTGGAGTCGGCAATTCAGTTGAGACAGTTCT3’ |
| miR-22 F1 | 5’ ACACTCCAGCTGGGAAGCTGCCAGTTGAAG 3’ |
| miR-361-RT | 5’CTCAACTGGTGTCGTGGAGTCGGCAATTCAGTTGAGGTACCCCT 3’ |
| miR-361 F1 | 5’ACACTCCAGCTGGGTTATCAGAATCTCCAG 3’ |
| miR-98-RT | 5’CTCAACTGGTGTCGTGGAGTCGGCAATTCAGTTGAGAACAATAC 3’ |
| miR-98 F1 | 5’ACACTCCAGCTGGGTGAGGTAGTAAGTTGT 3’ |
| miR-155-RT | 5’CTCAACTGGTGTCGTGGAGTCGGCAATTCAGTTGAGACCCCTAT3’ |
| miR-155 F1 | 5’ACACTCCAGCTGGGTTAATGCTAATCGTGAT 3’ |
| miR-99a -RT | 5’CTCAACTGGTGTCGTGGAGTCGGCAATTCAGTTGAGCACAAGAT 3’ |
| miR-99a F1 | 5’ACACTCCAGCTGGGAACCCGTAGATCCGAT 3’ |
| miR-223-RT | 5’CTCAACTGGTGTCGTGGAGTCGGCAATTCAGTTGAGTGGGGTAT 3’ |
| miR-223 F1 | 5’ACACTCCAGCTGGGTGTCAGTTTGTCAAAT 3’ |
| Universal reverse primer | 5’ CTCAACTGGTGTCGTGGA-3’ |
